# Dynamics of Actin Filaments Play an Important Role in Root Hair Growth under Low Potassium Stress in *Arabidopsis thaliana*

**DOI:** 10.3390/ijms25168950

**Published:** 2024-08-16

**Authors:** Mingyang Li, Shihang Liu, Jinshu Wang, Xin Cheng, Chengxuan Diao, Dabo Yan, Yue Gao, Che Wang

**Affiliations:** College of Bioscience and Biotechnology, Shenyang Agricultural University, Shenyang 110866, China; limingyang4396@126.com (M.L.); lsh19980506@163.com (S.L.); wangjinshu@stu.syau.edu.cn (J.W.); cylx0831@126.com (X.C.); chxuan2012@163.com (C.D.); 2002510009@syau.edu.cn (D.Y.)

**Keywords:** actin filaments, low K^+^ stress, VLN1, VLN4, *Arabidopsis*

## Abstract

Potassium (K) is an essential nutrient for the growth and development of plants. Root hairs are the main parts of plants that absorb K^+^. The regulation of plant root hair growth in response to a wide range of environmental stresses is crucially associated with the dynamics of actin filaments, and the thick actin bundles at the apical and sub-apical regions are essential for terminating the rapid elongation of root hair cells. However, the dynamics and roles of actin filaments in root hair growth in plants’ response to low K^+^ stress are not fully understood. Here, we revealed that root hairs grow faster and longer under low K^+^ stress than the control conditions. Compared to control conditions, the actin filaments in the sub-apex of fast-growing wild-type root hairs were longer and more parallel under low K^+^ stress, which correlates with an increased root hair growth rate under low K^+^ stress; the finer actin filaments in the sub-apex of the early fully grown Col-0 root hairs under low K^+^ stress, which is associated with low K^+^ stress-induced root hair growth time. Further, *Arabidopsis thaliana* actin bundling protein Villin1 (VLN1) and Villin4 (VLN4) was inhibited and induced under low K^+^ stress, respectively. Low K^+^ stress-inhibited VLN1 led to decreased bundling rate and thick bundle formation in the early fully grown phase. Low K^+^ stress-induced VLN4 functioned in keeping long filaments in the fast-growing phase. Furthermore, the analysis of genetics pointed out the involvement of VLN1 and VLN4 in the growth of root hairs under the stress of low potassium levels in plants. Our results provide a basis for the dynamics of actin filaments and their molecular regulation mechanisms in root hair growth in response to low K^+^ stress.

## 1. Introduction

Potassium (K) is a vital mineral nutrient for plant growth and development, playing roles in osmoregulation, enzyme activation, and nutrient transport [1,2,3,4]. The concentration of K^+^ in the cytoplasm of plants remains relatively stable at approximately 100 mM, but the concentration of K^+^ at the interface between roots and soil is generally within the range of 0.1 to 1 mM [5]. Therefore, in the natural environment, most plants usually suffer from low K^+^ stress. K^+^ deficiency is also a common abiotic stress that inhibits plant growth and development and reduces crop yield [6]. The root hair, which accounts for more than 77% of the total area of the roots, is an important organ that enables the plant to absorb water and nutrients during plant growth, development, and stress management [7,8]. When plants are in a state of nutrient deficiency, such as low potassium, it can affect the initial growth and development of lateral roots, inhibit the growth of taproots, and promote the elongation of root hairs. The roots activate two important adaptation mechanisms in order to absorb nutrients to maintain plant growth and survival. One adaptation is additional nutrient acquisition and remobilization systems. A high-affinity K^+^ uptake system plays a dominant role at low external K^+^ (below 0.2 mM), and some transporters such as K^+^ uptake 4 (KUP4), high-affinity K^+^ transporter 5 (HAK5), and so on also have an important function in it [9,10,11]. Another adaptation is changes in root development, such as root hair elongation [12].

The transcription factor RAP2.11, belonging to the AP2/ERF family, can specifically target the HAK5 promoter region under conditions of low K+ stress, exerting control over root growth [13]. Under K^+^ deficiency conditions, the ethylene signal interacts with zinc finger protein 5 (ZFP5) to regulate root hair elongation [14]. The crucial role played by the GL2-regulated-ETO1 module is evident in facilitating root hair growth under K^+^ deficiency conditions [15]. It can be seen that there are few studies on the mechanism of root hair growth under low K^+^ stress, and the underlying molecular mechanisms have remained unclear.

The dynamics of the cytoskeleton such as actin filaments have been proven to be an essential factor in regulating root hair growth [16,17,18]. *Arabidopsis* encodes three actin proteins (ACT2, ACT7, and ACT8), and double mutants *act2-1 act7-4* and *act8-2 act7-4* exhibit actin filament cytoskeleton abnormalities, while *act2-1 act8-2* shows complete inhibition of root hair growth [19]. Villin (VLN) is an important actin filament bundling protein in plants [20]. Given the biochemical functionalities exhibited by actin-binding proteins (ABPs), it is speculated that villin proteins (VLNs) play a key role in the arrangement of the thick bundles found in root hairs [17,21]. The *Arabidopsis* VLN family contains five isoforms (AtVLN1-AtVLN5) [20]. VLN1 was the initial plant villin protein to be characterized as possessing a function independent of calcium ions (Ca^2+^), and recombinant VLN1 binds to actin filaments with high affinity and produces a bundle filament network [22]. GUS staining analysis reveals that VLN1 is expressed at a high level in diverse plant tissues, such as leaves, hypocotyls, roots, and root hairs [20]. VLN3 has a similar ability to bind and bundle actin as VLN1 and possesses Ca^2+^-dependent severing activity [23]. VLN1 regulates the growth of root hair by regulating the binding of actin filaments in the apex and sub-apex of root hair, and its role is directly mediated by the transcription factor GL2 [24]. The recombinant VLN4 protein enables actin filament bundling, Ca^2+^-dependent filament severing, and barbed end capping [25]. Loss of function of VLN4 results in slower root hair growth [25]. The genetic analysis suggests that VLN2, VLN3, and VLN5 could potentially not be requisite for the growth and development of root hairs [24,26,27,28]. Moreover, several actin-binding proteins have been found to be associated with root hair growth in *Arabidopsis* [8,17,29,30]. There are significant changes in the root hair growth of actin depolymerizing factor 1 (ADF1) genetic material [17]. Microtubule-associated protein MAP18 can interact with ROP2 to affect root hair development [29]. Actin-depolymerizing protein ADF7 promotes root hair formation by inhibiting the expression and function of VLN1 [8]. Furthermore, it has been shown in a recent investigation that under high pH conditions, actin depolymerizing factors 8 and 11 play a role in the extension of root hairs [30]. However, the dynamics and roles of actin filaments in root hair growth in plants responding to low K^+^ stress are completely unknown.

In this study, we revealed that the actin filaments in the sub-apex of fast-growing wild-type Col-0 root hairs had longer and more parallel growth under low K^+^ stress. In the early fully grown phase, there were no significant visible thick bundles in the apex in Col-0 hairs under low K^+^ stress. These results indicated that low K^+^ could prolong root hair growth time and increase growth rate. Moreover, low K^+^ stress-inhibited VLN1 led to decreased bundling and thick bundle formation in the early fully grown phase, and low K^+^ stress-induced VLN4 led to the increasing single filament lifetime and single filament length in the fast-growing phase. Our results suggest that dynamics of actin filaments are required for root hair growth in *Arabidopsis* tolerance of low K^+^ stress.

## 2. Results

### 2.1. Low K^+^ Stress Prolongs Root Hair Growth Time and Improves Root Hair Growth Rate

**Figure 1:**
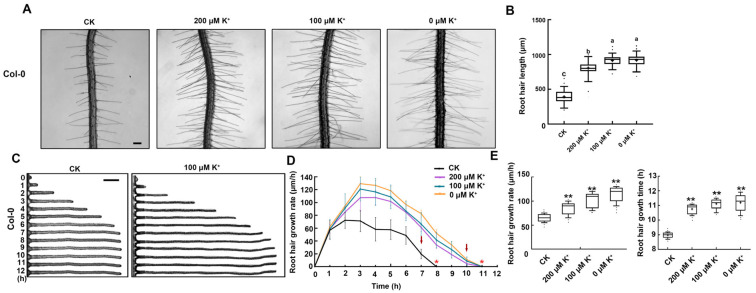
Low K^+^ stress prolongs root hair growth time and improves growth rate. (**A**) Images of root hairs of Col-0 under the CK condition and low K^+^ treatments. Scale bar, 200 μm. (**B**) The length of root hairs in Col-0 under the CK condition and low K^+^ treatments. Significant differences for each state are signified by lower-case characters through one-way ANOVA combination with Tukey’ s test (*p* < 0.05). (**C**) The growth rate of Col-0 root hairs under the CK condition and 100 μM K^+^ treatment. Scale bar, 100 μm. (**D**) The average growth rates of root hairs, measured in micrometers per hour, in Col-0 under the CK condition and low K^+^ treatments. The arrows over polylines indicate the moments when the growth rate drops significantly, which is at 7 h for Col-0 under the CK condition and 10 h for Col-0 under low K^+^ treatment. The presence of asterisk symbols over the polylines indicates the commencement of the fully grown phase, with the time being at 8 h for Col-0 under the CK condition and 11 h for Col-0 under low K^+^ treatments. Values represent means ± SE. (**E**) Images of root hair growth rate and root hair growth time in Col-0 under the CK condition and low K^+^ treatments. Asterisk symbols denote a statistically significant difference in comparison with CK, as evaluated by Student’s *t*-test (** *p* < 0.01). (**A**,**B**) reveal the mean lengths of root hairs situated in the region ranging from 2 to 4 mm away from the root apex of six-day-old seedlings (more than 500 hairs in no fewer than 50 individual roots). In (**C**,**D**), the growth of root hairs was quantified starting from the bulges up to the 12 h time point of four-day-old seedlings (more than 100 hairs in no fewer than 30 individual roots).

According to the report, K^+^ deficiency can promote the growth of root hair in plants [14]. To further study the dynamics of root hair growth under low K^+^, we first examined the average root hair length of *Arabidopsis* Columbia (Col-0) under control conditions (CK, 2.5 mM K^+^) and low K^+^ (200 μM, 100 μM, and 0 μM K^+^) treatments (Figure 1A). The average root hair length of Col-0 was ~390 μm under the CK condition (Figure 1A,B). Compared with the control, Col-0 root hairs under low K^+^, including 200 μM K^+^, 100 μM K^+^, and 0 μM K^+^ treatments, were longer at ~793 μm, ~849 μm, and ~939 μm, respectively (Figure 1A,B). Low K^+^ treatments significantly increased the root hair length, and there were significant differences between low K^+^ treatments (Figure 1A,B). These results prove that low K^+^ can induce root hair growth. Then, the rate at which root hairs grew in Col-0 plants under CK and low K^+^ treatments was measured at 1 h intervals over a 12 h period, beginning with the initiation of bulge formation (Figure 1C). In the CK condition, Col-0 hairs exhibited a rapid growth rate ranging from 45 to 70 μm h^−1^ during the initial 6 h. However, by 7 h, a significant decrease in the growth rate was observed, indicating that they had entered the terminating-growth phase. By 9 h, they hardly grew any longer, signifying their entrance into the fully grown phase (Figure 1C,D). Compared with the control, Col-0 root hairs with low K^+^ treatment displayed a significantly increased growth rate from the second h, continued to grow fast until 10 h, then precipitously slowly grew and almost stopped growth by 11 h (Figure 1C,D). In the fast-growing phase, low K^+^ treatment increased the growth rate of Col-0 root hairs to 120 μm h^−1^ (Figure 1E). Low K^+^ treatment increased the growth time of Col-0 root hairs by 2 h (Figure 1E). These results indicated that low K^+^ could prolong root hair growth time and increase growth rate.

### 2.2. Dynamics of Actin Filaments Are Affected in Root Hair Growth under Low K^+^ Stress

**Figure 2:**
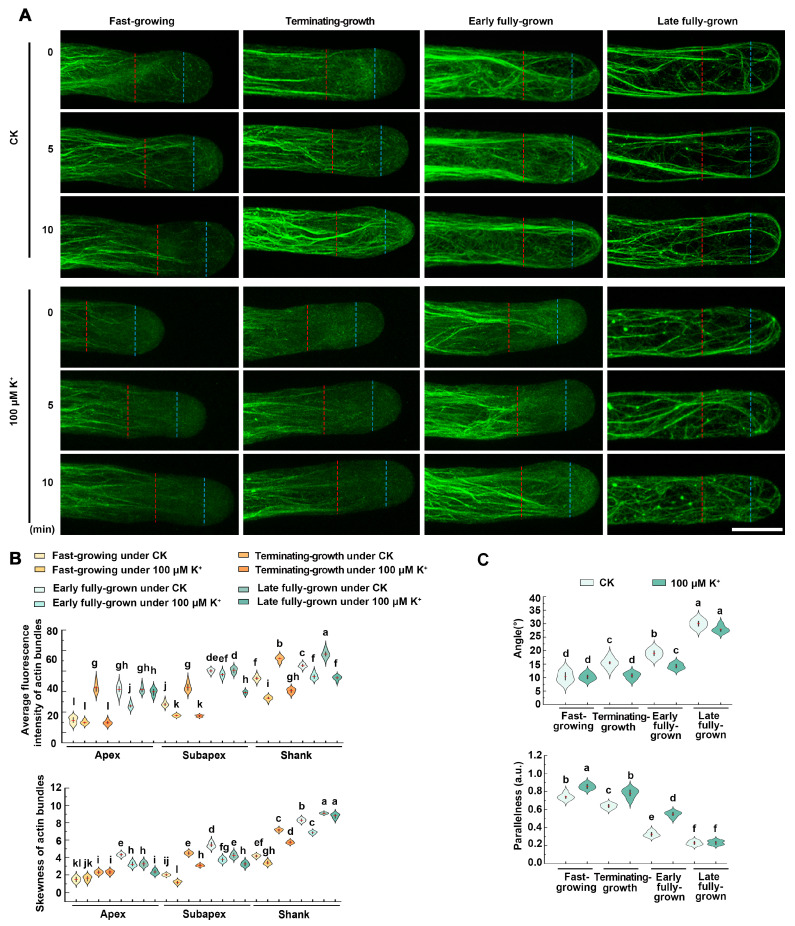
Low K^+^ stress affects the dynamics of actin filaments in root hair growth. (**A**) Time-lapse images of actin filaments during the growth of Col-0 root hairs under the CK condition and 100 μM K^+^ treatment at the specific time points of 0, 5, and 10 min of the diverse growth stages. The region between the tip of the root hairs and the blue line represents the apex; the region between the blue and red lines represents the sub-apex; the region behind the red line represents the shank. Scale bar, 10 μm. (**B**) Average fluorescence intensity and skewness of actin bundles of root hairs during diverse growth stages in Col-0 under the CK condition and 100 μM K^+^ treatment. (**C**) Angle and parallelness of root hairs during diverse growth stages in Col-0 under the CK condition and 100 μM K^+^ treatment. The fluorescence intensity was determined by gauging the fluorescence pixel intensity in the sub-apex and apex regions of root hairs (more than 50 hairs in no fewer than 30 individual roots). The measurement of the average fluorescence intensity of actin bundles and skewness was conducted on the images by employing ImageJ. The values signify the mean along with the ± SE. Lower-case characters denote significant differences by one-way ANOVA combination with Tukey’ s test (*p* < 0.05).

The actin cytoskeleton is vital for the process of root hair elongation [17,18]. To investigate whether the dynamics of actin filaments are affected during the growth of root hair under low K^+^ stress, we used *f*ABD2-GFP, an ideal actin filament-specific fluorescent probe, to observe actin filament dynamics in the apex and sub-apex regions of root hair cells in Col-0 under CK and 100 μM K^+^ treatments. As shown in Figure 2A, we discovered that during the fast-growing phase, the Col-0 root hairs under the CK condition exhibited no significant visible filaments at the apex of root hairs, and several fine filament bundles aligned along the growth axis in the sub-apex. Meanwhile, the Col-0 root hairs under low K^+^ treatment showed a larger space of the absent filaments in the apex and longer and finer actin filaments with more parallel growth axes in the sub-apex. This finding was in line with the fact that the average fluorescence intensity and skewness (the extent of actin filament bundling) parameter in the sub-apex and the apex were lower under low K^+^ treatments than the CK condition (Figure 2B). In the terminating-growth phase, compared with the fast-growing phase, Col-0 root hairs under the CK condition possessed several bundles appearing in the apex and several brighter and thicker bundles with larger angles in the sub-apex (Figure 2A). This result was in line with the fact that average fluorescence intensity and skewness parameter in the sub-apex of Col-0 hairs were higher in the terminating-growth phase compared with the fast-growing phase (Figure 2B). The actin filaments of Col-0 root hairs under low K^+^ treatment in the terminating-growth phase were similar to those under the CK condition in the fast-growing phase (Figure 2A). In the early fully grown and late fully grown phases, the brighter and thicker actin filaments of Col-0 root hairs under the CK condition appeared to spiral and extended to the top (Figure 2A). During the early fully grown phase, actin filaments of the Col-0 root hairs under low K^+^ treatment were similar to that under CK conditions in the terminating-growth phase (Figure 2A). Bundles looped through the extreme apex during the late fully grown phase, and there was no significant difference in actin filaments of Col-0 root hairs between CK and low K^+^ treatments (Figure 2A,B). In conclusion, during the fast-growing phase, under low K^+^ treatment, the actin filaments in the sub-apex and larger space of the absent filaments in the apex were larger than those in the CK condition, which were similar to those in the terminating-growth and early fully grown phases (Figure 2A,B).

In addition, we also quantified the average filament angle relative to the root hair growth axis (angle), and the parallelness of filaments to each other (Figure 2C). In the fast-growing, the terminating-growth, and the early fully grown phases, actin filaments showed lower average filament angles and higher parallelness under low K^+^ treatment than the CK condition (Figure 2C). These findings indicated that compared with the CK condition, actin filaments were more parallel to the root hair growth axis under low K^+^ treatment.

### 2.3. The Expression of VLN1 and VLN4 in Root Hairs Is Affected under Low K^+^ Stress

**Figure 3:**
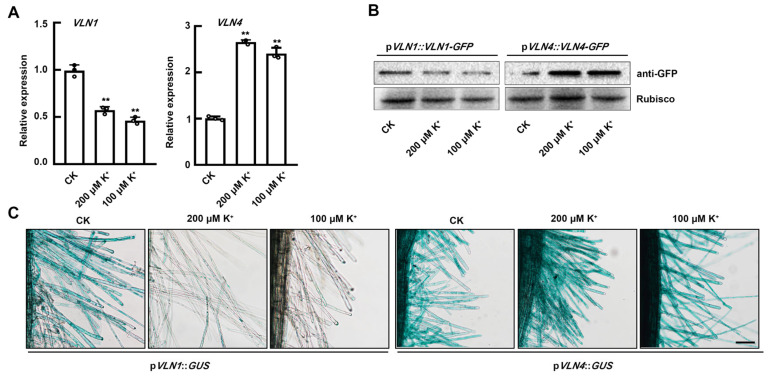
Low K^+^ stress affected the expression of *VLN1* and *VLN4* in root hairs. (**A**) qRT-PCR quantification of *VLN1* and *VLN4* expression level in Col-0 under the CK condition and low K^+^ treatments. Data signify the average ± SD of three replicates. Asterisk symbols denote a statistically significant difference in comparison with Col-0 under the CK condition, as ascertained by Student’s *t* test (** *p* < 0.01). (**B**) Western blotting of VLN1 and VLN4 expression level in Col-0 seedlings under the CK condition and low K^+^ treatments. Rubisco as a loading control. (**C**) GUS assay of *VLN1* and *VLN4* expression in root hairs from Col-0 seedling under the CK condition and low K^+^ treatments. Scale bar, 100 μm.

Previous studies reported that VLN1 and VLN4 can regulate root hair growth [8,24,25]. To investigate whether VLN1 and VLN4 are related to the regulation of *Arabidopsis* root hair growth by potassium deficiency, we first identified whether these genes are induced by low K^+^ stress. The result of qRT-PCR showed that *VLN1* expression was significantly down-regulated and *VLN4* expression was significantly up-regulated in response to low K^+^ stress (Figure 3A). This was further verified by Western blotting analysis (Figure 3B). In addition, *pVLN1::GUS* transgenic lines and *pVLN4::GUS* transgenic lines were also used to determine whether *VLN1* and *VLN4* respond to low K^+^ stress. After low K^+^ treatment, the promoter activity of *pVLN1* in root hairs was decreased (Figure 3C). By contrast, the promoter activity of the *pVLN4::GUS* transgenic seedlings was increased with low K^+^ treatments (Figure 3C). The results from qRT-PCR, Western blotting, and GUS analysis demonstrated that the application of low K+ treatments caused a reduction in the expression of VLN1 and an enhancement in the expression of VLN4 within root hairs.

### 2.4. VLN1 and VLN4 Regulate Root Hair Growth under Low K^+^ Stress

To further determine the role of VLN1 and VLN4 in low-K^+^- stress-mediated root hair growth, we first identified three T-DNA insertion mutants: *vln1-1* (SALK_020027), *vln1-2* (SALK_133579), and *vln4-1* (SALK_049058). We also constructed two complementation lines (*VLN1* comp9 and *VLN1* comp14) by transforming *VLN1* promoter *pVLN1::VLN* in *vln1-1* and *vln1-2* plants, and two complementation lines (*VLN4* comp7 and *VLN4* comp10) by transforming *VLN4* promoter *pVLN4::VLN4* in *vln4-1* plants (Figure S1).

**Figure 4:**
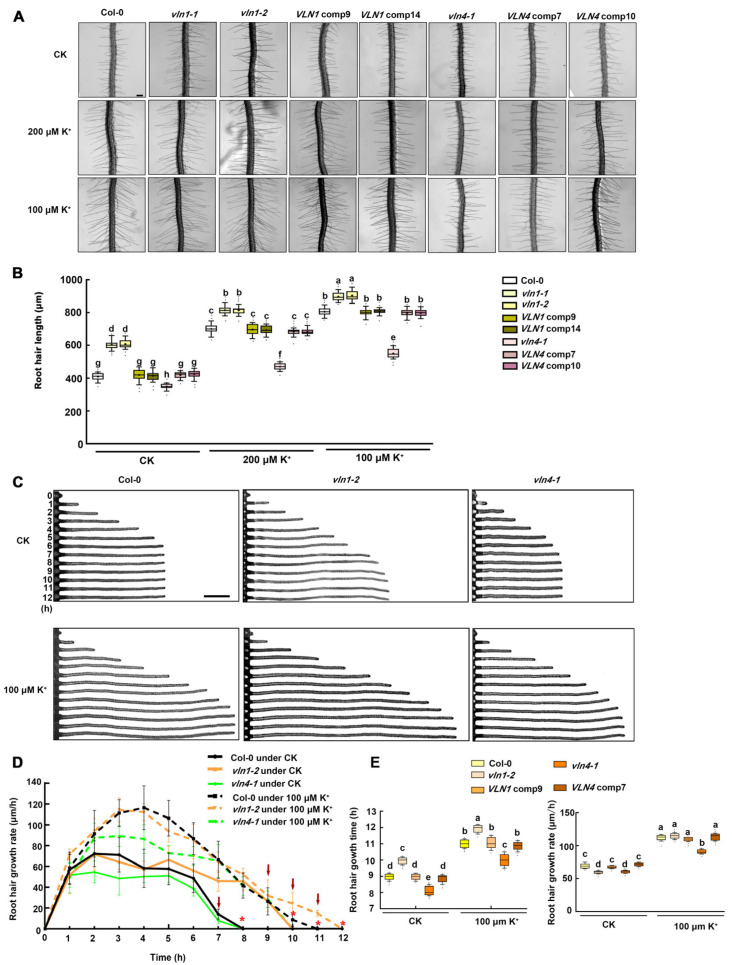
The roles of VLN1 and VLN4 are involved in low K^+^ stress-mediated root hair growth. (**A**) Images of root hairs under the CK condition and low K^+^ treatments from the Col-0, different genotypes of VLN1 and VLN4. Scale bar, 200 μm. (**B**) The length of root hairs from different genotypes of VLN1 and VLN4 under the CK condition and low K^+^ treatments. Significant differences, as identified by one-way ANOVA combination with Tukey’s test (*p* < 0.05), are signified by lower-case characters. (**C**) Images of root hair growth from Col-0, *vln1-2*, and *vln4-1* over time under the CK condition and 100 μM K^+^ treatment. Scale bar, 100 μm. (**D**) The average of root hair growth rates expressed in micrometers per hour for Col-0, *vln1-2*, and *vln4-1* under the CK condition and 100 μM K^+^ treatment. The arrows over polylines indicated the moments when the growth rate drops significantly, which was at 7 h for Col-0 and *vln4-1* under the CK condition, 9 h for *vln1-2* under the CK condition and *vln4-1* under 100 μM K^+^ treatment, 10 h for Col-0 under 100 μM K^+^ treatment, and 11 h for *vln1-2* under 100 μM K^+^ treatment. The presence of asterisk symbols over the polylines indicates the commencement of the fully grown stages, with the time being at 9 h for Col-0 and *vln4-1* under the CK condition, 10 h for *vln1-2* under the CK condition and *vln4-1* under 100 μM K^+^ treatment, 11 h for Col-0 under 100 μM K^+^ treatment, and 12 h for *vln1-2* under 100 μM K^+^ treatment. Values represent means ± SE. (**E**) Images of root hair growth rate and root hair growth time in Col-0, *vln1-2*, *VLN1* comp9, *vln4-1*, and *VLN4* comp7 under the CK condition and 100 μM K^+^ treatment. (**A**,**B**) reveal the mean lengths of root hairs situated in the region, ranging from 2 to 4 mm away from the root apex of four-day-old seedlings (more than 500 hairs in no fewer than 50 individual roots). In (**C**,**D**), the growth of root hairs was quantified starting from the bulges up to the 12 h time point of four-day-old seedlings (more than 100 hairs in no fewer than 30 individual roots).

Then, we determined the length and growth rate of root hairs in Col-0, *vln1-1*, *vln1-2*, *vln4-1*, *VLN1* comp9, *VLN1* comp14, *VLN4* comp7, and *VLN4* comp10 seedlings following low K^+^ treatment at various concentrations (Figure 4). In contrast to Col-0, *vln1-1* and *vln1-2* exhibited longer hairs, but *vln4-1* seedlings had shorter hairs under CK conditions and low K^+^ treatments (Figure 4A,B). Root hairs length was rescued to Col-0 level in *VLN1* comp9, *VLN1* comp14, *VLN4* comp7, and *VLN4* comp10 plants (Figure 4A,B). Furthermore, compared to Col-0, the growth rate of *vln1-2* was similar in the fast-growing phase as well as the terminating-growth phase and maintained root hair growth in the early fully grown phase under CK and 100 μM K^+^ treatment, resulting in 3 h longer growth time under CK and 1 h longer growth time under 100 μM K^+^ treatment, and *vln4-1* hairs showed slow growth rate during the fast-growing phase under CK and 100 μM K^+^ treatment, especially under 100 μM K^+^ treatment, and similar growth time to Col-0 hairs under CK and 100 μM K^+^ treatment (Figure 4C–E). These results showed that VLN1 negatively regulated the root hair growth time and VLN4 positively regulated the root hair growth rate under low K^+^ stress.

### 2.5. VLN1 and VLN4 Regulate Dynamics of Actin Filaments in Root Hair Growth under Low K^+^ Stress

**Figure 5:**
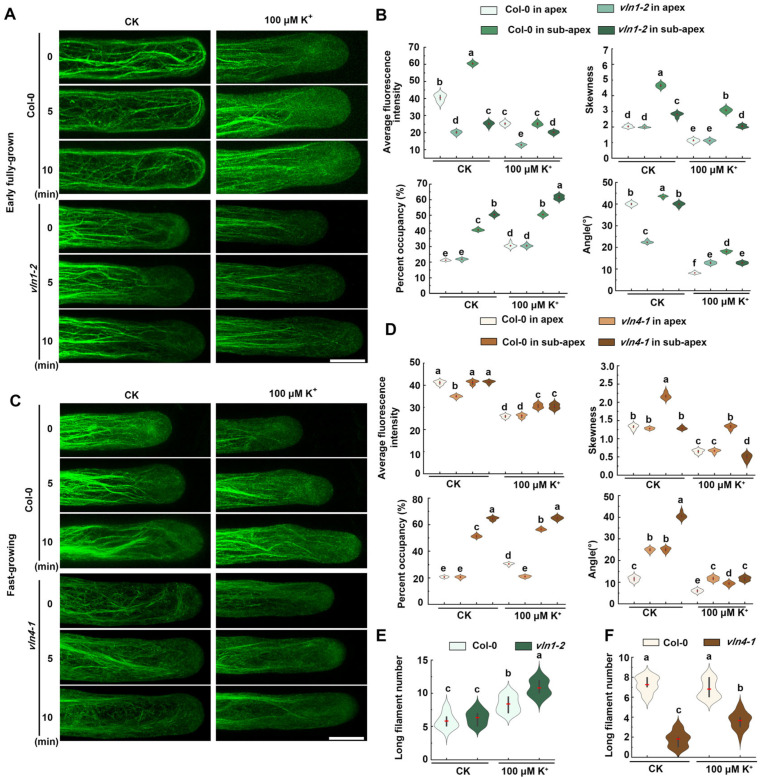
Actin filament dynamics are regulated by VLN1 and VLN4 in root hairs under low K^+^ stress. (**A**) Time-lapse images of actin filaments at 0, 5, and 10 min during the early fully grown phase of root hair growth of Col-0 and *vln1-2* under the CK condition and 100 μM K^+^ treatment. Scale bar, 10 μm. (**B**) Mean fluorescence intensity, percentage occupancy, skewness, and angle of root hairs in Col-0 and *vln1-2* during the early fully grown phase under the CK condition and 100 μM K^+^ treatment. (**C**) Time-lapse images of actin filaments in the growth of root hairs of Col-0 and *vln4-1* at 0, 5, and 10 min in the fast-growing phase under the CK condition and 100 μM K^+^ treatment. Scale bar, 10 μm. (**D**) The level of average fluorescence intensity, percentage occupancy, skewness, and angle of root hairs in Col-0 and *vln4-1* during the fast-growing phase under the CK condition and 100 μM K^+^ treatment. (**E**) Long filament number of root hairs in Col-0 and *vln1-2* in the early fully grown phase under the CK condition and 100 μM K^+^ treatment. (**F**) Long filament number of root hairs in Col-0 and *vln4-1* in the fast-growing phase under the CK condition and 100 μM K^+^ treatment. More than 50 hairs in no fewer than 30 individual seedlings were calculated in (**B**,**D**). The values signify the mean along with the ± SE. Significant differences of different genotypes and treatments were indicated by lower-case characters letters through the application of one-way ANOVA combination with Tukey’s test (*p* < 0.05).

To explore the actin filament dynamic mechanisms of VLN1 and VLN4 during root hair growth under low K^+^ stress, we observed the actin filament dynamics of *vln1* and *vln4* seedlings during root hair growth (Figure 5). Because VLN1 mainly plays a role in the early fully grown phase under CK conditions and low K^+^ stress, we mainly observed the effect of VLN1 on the dynamics of actin filaments in the early fully grown phase. Our results showed that, in the early fully grown phase, compared with Col-0, *vln1-2* root hairs under low K^+^ stress maintained finer bundles, and most of them were growth-axially aligned fine bundles (Figure 5A). This result was consistent with the reduction in average fluorescence intensity and the lower skewness in the sub-apex, together with the increased percentage of occupancy in the apex and sub-apex of *vln1-2* under low K^+^ stress (Figure 5B). Because VLN4 mainly plays a role in the fast-growing phase, we mainly observed the effect of VLN4 on the dynamics of actin filaments in the fast-growing phase. We found that *vln4-1* maintained finer filaments in the fast-growing phase of root hair under the CK condition and low K^+^ stress, compared with Col-0 (Figure 5C,D). The angle between the actin filaments and the growth direction of root hair increased under CK condition, but it was not obvious under low K^+^ stress (Figure 5C,D). In Figure 5C, it is obvious that *vln4* root hairs displayed fewer long filaments than Col-0. Therefore, we counted the long filament number, and we found that long filaments decreased significantly in *vln4* root hairs under CK conditions and low K^+^ stress (Figure 5F).

**Figure 6:**
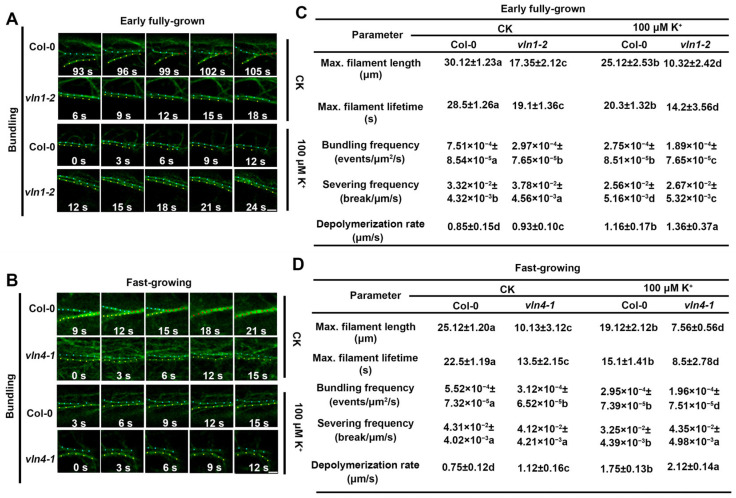
The single actin filament dynamics are regulated by VLN1 and VLN4 in root hairs under low K^+^ stress. (**A**) Bundling processes in Col-0 and *vln1-2* seedlings in the early fully grown phase under the CK condition and 100 μM K^+^ treatment. Scale bar, 10 μm. (**B**) Bundling processes in Col-0 and *vln4-1* seedlings in the fast-growing phase under the CK condition and 100 μM K^+^ treatment. Scale bar, 10 μm. (**C**) In the early fully grown phase, the parameters of actin dynamics are regulated by VLN1 on the single actin filament level in root hairs under the CK condition and 100 μM K^+^ treatment. (**D**) In the fast-growing phase, the parameters of actin dynamics are regulated by VLN4 on the single actin filament level in root hairs under the CK condition and 100 μM K^+^ treatment. For the analysis of the bundling frequency, a region of 30 × 30 μm^2^ was selected. More than 60 hairs in no fewer than 20 individual seedlings were calculated. The values signify the mean along with the ± SE. Significant differences of different genotypes and treatments were indicated by lower-case characters letters through the application of one-way ANOVA combination with Tukey’s test (*p* < 0.05).

Both VLN1 and VLN4 are actin filament bundling proteins [24,25]. Therefore, we further explored the single actin filament bundling capacities of VLN1 and VLN4 in root hairs. The results suggested that during the early fully grown phase, in contrast to Col-0, *vln1-2* under the CK condition led to a decline in single actin filament bundling frequency (Figure 6A,C). Similarly, compared with Col-0, *vln4-1* under the CK condition also led to a decline in single actin filament bundling frequency during the fast-growing phase (Figure 6B,D). Moreover, compared with CK conditions, the single actin filament bundling frequency of Col-0 and *vln1-2* under low K^+^ stress was significantly reduced in the early fully grown phase and *vln4-1* in the fast-growing phase, respectively (Figure 6C,D). These results suggested that VLN1 and VLN4 respond to low K^+^ stress by affecting the dynamic changes of actin filament bundling in the root hair growth. Because the change of single actin filament bundling capacities may affect the other single actin filament dynamic, we also observed the single actin filament dynamic changes of Col-0, *vln1-2*, and *vln4-1* under the CK condition and low K^+^ stress (Figure 6C,D). The results showed that compared with CK conditions, under low K^+^ stress, the maximum filament length, maximum filament lifetime, and single actin filament severing frequency of Col-0 decreased in both early fully grown and fast-growing phases, while the rate of single actin filament depolymerization increased; compared with Col-0, *vln1-2* in the early fully grown phase and *vln4-1* in the fast-growing phase had a decline in maximum filament length and maximum filament lifetime and an increase in single actin filament severing frequency and depolymerization rate under the CK condition and low K^+^ stress (Figure 6C,D). Additionally, under low K^+^ stress, *vln1-2* showed a lower single actin filament severing frequency in the early fully grown phase than that under CK conditions, and the single actin filament severing frequency of *vln4-1* under low K^+^ stress in the fast-growing phase was higher than that under CK conditions (Figure 6C,D). The above results indicated that there were differences in the dynamics of some single actin filaments between VLN1 and VLN4.

### 2.6. VLN1 and VLN4 Affect the Seedlings Growth under Low K^+^ Stress

**Figure 7:**
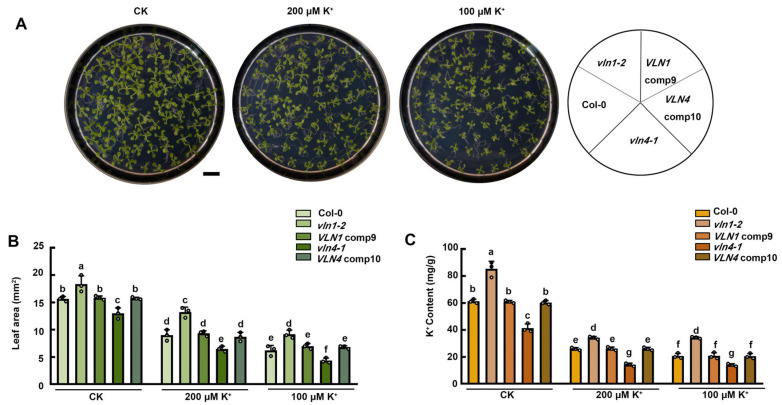
Effects of VLN1 and VLN4 on plant growth under low K^+^ stress. (**A**) Images of growth state from 16-day-old seedlings of Col-0, *vln1-2*, *vln4-1*, *VLN1* comp9, and *VLN4* comp10 under the CK condition and low K^+^ treatments. Scale bar, 1 cm. (**B**,**C**) Leaf area and K^+^ content from 16-day-old seedlings of Col-0, *vln1-2*, *vln4-1*, *VLN1* comp9, and *VLN4* comp10 under the CK condition and low K^+^ treatments. The values signify the mean along with the ± SE. Significant differences of different genotypes and treatments were indicated by lower-case characters letters through the application of one-way ANOVA combination with Tukey’s test (*p* < 0.05).

We also investigated the effect of VLN1 and VLN4 on seedling growth with low K^+^ stress. The growth phenotypes and K^+^ contents of Col-0, *vln1-2*, *vln4-1*, *VLN1* comp9, and *VLN4* comp10 seedlings were analyzed under low K^+^ stress (Figure 7A,C). Compared with Col-0, *vln1-2* mutants displayed larger leaf areas and higher K^+^ contents under low K^+^ treatments, whereas *vln4-1* seedlings showed contrary results (Figure 7B,C). There was no significant difference in leaf area and K^+^ content between *VLN1* comp9 and *VLN4* comp10, compared with Col-0 seedlings (Figure 7B,C). These results indicated that VLN1 plays a role in reducing the ability of plants to tolerate low K^+^, while VLN4 improves the ability of plants to tolerate low K^+^. This is consistent with the effect of VLN1 and VLN4 on regulating root hair growth under low K^+^ stress.

## 3. Discussion

Plant growth and development rely on the vital nutrient K^+^, prompting plants to adjust metabolically and morphologically under nutrient-deficient conditions [14]. In response to K^+^ stress, plants promote nutrient uptake by increasing root hair length and root surface area [12,31,32]. The dynamics of actin filaments have been proven to be an essential factor in regulating root hair formation and growth [16,17,18]. However, the dynamics of actin filaments in root hair growth in plants response to low K^+^ stress are fully unknown.

At present, the root hair growth process under low K^+^ stress has not been comprehensively and carefully observed. We found that the root hair grew faster under low K^+^ stress than under CK conditions from the second hour, and this rapid growth was maintained for eight hours (Figure 1). Meanwhile, the growth time of root hairs under low K^+^ stress was increased by two hours compared with CK conditions (Figure 1). Our results provided a detailed analysis of the root hair growth process under low K^+^ stress, which serves as solid evidence for our subsequent research and other related studies on root hair growth under low K^+^ stress.

At various stages of root hair growth and development, the actin cytoskeleton is involved by organizing into different arrangements [17]. However, the dynamic changes of actin filaments in root hair growth during low K^+^ stress are still unclear. We found that in the fast-growing phase, Col-0 root hairs under the CK condition possessed no significant visible filaments in the apex of root hairs and several growth-axially aligned fine filament bundles in the sub-apex (Figure 2). In the terminating-growth phase, several fine bundles appear in the apex and several brighter and thicker bundles in the sub-apex (Figure 2A). In the early fully grown and late fully grown phases, the brighter and thicker actin filaments appeared to spiral and extended to the top (Figure 2). These results were consistent with the previous reports [17,24,33]. Compared with the CK condition, in the fast-growing phase, Col-0 root hairs under low K^+^ treatment showed a larger space of the absent filaments in the apex and longer and finer actin filaments with more parallel growth axes in the sub-apex (Figure 2). In the terminating-growth phase and the early fully grown phase, the actin arrays under low K^+^ treatment were similar to those in the fast-growing phase and the terminating-growth phase under CK condition, respectively (Figure 2). These results were consistent with the longer growth time of root hairs under low K^+^ treatments. In the late fully grown phase, several thick bundles looped through the extreme apex, and there was no significant difference in actin filaments of Col-0 root hairs between the CK and low K^+^ treatments (Figure 2A). In addition, we found that the actin filaments are more parallel to the root hair growth axis in various root hair growth phases under low K^+^ treatment (Figure 2). This may be related to the rapid material transport, because long bundles of actin filaments function as routes for vesicle and organelle movement [17,24,33]. Therefore, the more parallel actin array may be helpful to the root hair growth, which may be a major reason for the increased growth rate of root hairs under low K^+^ stress. Moreover, recent research indicates the significance of ADF8 and ADF11 within the ADF family in maintaining the intricate structure of actin filaments at the apex of root hairs [30]. Meanwhile, the morphology of actin filaments is influenced by the dynamics of multiple single actin filaments, and different ABPs have different abilities to regulate the dynamics of single microfilaments. Thereby, other ABPs may also be involved in the process of low K^+^-induced parallel filaments, and such results provide a more comprehensive elucidation for the interpretation of the dynamic process and function of single microfilaments in the root hair growth process under low K^+^ stress. Consequently, we will investigate which ABPs are involved in the low K^+^-induced root hair growth process in the future. Compared with CK conditions, the actin filaments of root hairs under low K^+^ stress were shown to be significantly thinner, suggesting that their bundling capability was seriously affected under low K^+^ stress (Figure 2). Previous reports have found that villin is one of the major proteins responsible for organizing actin filaments into bundles [34,35]. Among the five members of the villin family, it has been demonstrated that VLN1 negatively participates in root hair growth and VLN4 positively participates in root hair growth [24,25]. Therefore, we speculate that VLN1 and VLN4 may be involved in the process of root hair growth induced by low K^+^ stress. Our analysis of RT-qPCR, GUS staining, and Western blotting illustrated that VLN1 and VLN4 respond negatively and positively to low K^+^ stress in root hairs, respectively (Figure 3). This is consistent with their function under normal conditions [24,25]. Therefore, it indicates that they are highly likely involved in the root hair growth process induced by low K^+^ stress. Further, we observed the length and growth of root hairs in *vln1* and *vln4* mutants under low K^+^ stress. Our results showed that compared with Col-0, *vln1-1* and *vln1-2* had longer hairs but *vln4-1* seedlings had shorter hairs under the CK condition which is consistent with previous reports (Figure 4) [24,25], and the root hairs of *vln1* and *vln4* were longer and shorter than those of Col-0 seedlings under low K^+^ stress, respectively (Figure 4 A,B). Further analysis showed that VLN1 mainly affected root hair growth time while VLN4 regulates root hair growth rate under low K^+^ stress (Figure 4C–E).

Wang et al. found that VLN1 is necessary to bundle sub-apical and apical actin filaments into thick bundles during the early fully grown phase [24]. Our observations of actin filament dynamics under CK conditions are consistent with those previously reported (Figure 5). Under low K^+^ stress, the *vln1* mutant displayed thinner actin filaments, fewer thick actin filament bundles, and a lower bundling in the early fully grown phase (Figure 5A,B), suggesting that VLN1 affects actin bundling in the early fully grown phase. Zhang et al. found that the root hairs of the *vln4* mutant became shorter and the growth rate was slower, which may be due to the loss of VLN4 function leading to changes in cytoplasmic streaming routes and rates, as well as a decrease in axial and apical actin bundles; however, the dynamic processes of actin filaments during the fast-growing phase of the *vln4* mutant has not been observed in detail [25]. We found that under low K^+^ stress, the loss of function of *VLN4* led to a reduction in the number of the thick bundles of actin filaments in root hairs and decreased bundling of the signal filaments during the fast-growing phase (Figure 5C,D), suggesting that VLN4 affects actin bundling in the fast-growing phase.

In this study, the main functions of VLN1 and VLN4 induced by low K^+^ stress have been revealed. However, whether they possess other ancillary functions remains to be investigated meticulously. There are certain differences in the length of root hairs between wild types of other backgrounds and Col-0 in *Arabidopsis*. Additionally, root hairs grown on different culture media also showed certain variations. Hence, in other comparable studies, the lengths of the controlled root hairs might differ slightly. In this research, the wild-type (Col-0) and the *vln1* and *vln4* mutants are all of the Columbia background, and the culture medium employed is a regular brand product. Consequently, our conclusion is scientifically warranted.

## 4. Conclusions

**Figure 8:**
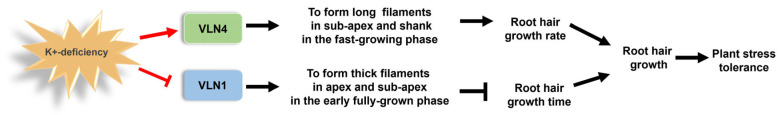
Working model of VLN1 and VLN4 in root hair growth in response to low K^+^ stress in *Arabidopsis*.

In summary, we have identified the dynamics of root hair growth and the dynamics of actin filaments during root hair growth under low K^+^ stress, and VLN1 and VLN4 participate in this process (Figure 1, Figure 2, Figure 3, Figure 4, Figure 5, Figure 6 and Figure 7). Low K^+^ stress inhibits *VLN1* expression, leading to thinner bundles in the early fully grown phase to promote root hair growth time, and low K^+^ stress increases *VLN4* expression, leading to keeping the long actin filaments in the fast-growing phase to improve root hair growth rate (Figure 8). Our results illustrate that actin filaments play an important role in root hair growth under low K^+^ stress and provide the dynamics of actin filaments and their molecular regulation mechanisms in low K^+^ stress-induced root hair growth.

## 5. Materials and Methods

### 5.1. Plant Growth Conditions and the Measurement of Growth Status

*Arabidopsis thaliana* Columbia (Col-0) ecotype was used as the wild type in this study. The *Atvln1-1*, *Atvln1-2*, and *Atvln4-1* mutant plants were respectively obtained from the seed stocks of the SALK_020027, SALK_133579, and SALK_049058 lines. Seeds were sterilized and incubated at 4 °C for 3 d and then sown on one-half strength MS (1/2 MS) medium (pH 5.8) with 0.8% (*w/v*) agar. The plants were grown in a growth chamber at 22 °C under a photoperiod of 16-hours-light and 8-hours-dark. For low K^+^ treatments, MgSO_4_·7H_2_O (1.5 mM), Ca(NO_3_)_2_·4H_2_O (2.99 mM), and NH_4_H_2_PO_4_ (1.25 mM) were applied to substitute for all the macro elements of 1/2 MS medium, and KCl (200 μM, 100 μM, and 0 μM) was used to adjust K^+^ content. Seedlings aged 3 days were transferred to a growth medium containing 100 μM and 200 μM of K^+^ for 13 days. Subsequently, a minimum of 60 plants with three technical and biological replicates were subjected to testing at the age of 16 days. The leaf area was investigated and photographed and then counted by ImageJ. All results were tested at three technical and biological replicates.

### 5.2. Plasmid Construction and Plant Transformation

The promoter of *VLN1* (*pVLN*1) and *VLN4* (*pVLN4*) was inserted into the *pCAMBIA1300-221* vector to create *pVLN1*::GUS and *pVLN4*::GUS. The CDS of *VLN1* and *VLN4* were inserted into the *pCAMBIA1205-GFP* vector to create *pVLN1::VLN1-GFP* and *pVLN4::VLN4-GFP*. We constructed *VLN1* and *VLN4* complementation lines (comps) by transforming the *VLN1* and *VLN4* promoter in *vln1-1*, *vln1-2*, and *vln4-1* plants, respectively. Primers used for plasmid construction are listed in Table S1. Following the construction process, *Agrobacterium tumefaciens* strain GV3101 was utilized to transfer all plasmids into *Arabidopsis* Col-0 using the floral dip technique. Subsequent generations of genetically modified plants underwent screening on 1/2 MS medium supplemented with hygromycin until achieving homozygosity.

### 5.3. Observation and Analysis of Root Hair Growth

The seedlings were cultivated on the 1/2 MS medium for 3 d and subsequently transferred to the low K^+^ (0 μM, 100 μM, and 200 μM K^+^) medium for another 3 d before being used for the observation and analysis of root hairs. Root hairs situated between 2 and 4 mm from the primary root tip of four-day-old seedlings in the CK condition and low K^+^ treatments were observed using a stereoscopic microscope (SMZ-168, MOTIC) following the previously reported method [15]. The length of root hairs within the same focal plane was determined through the analysis of seedling images processed with ImageJ. For these parameters, the root hair length of 30 individual seedlings of each genotype was evaluated in more than 500 root hairs, and the growth rate of 30 individual seedlings of each genotype was evaluated in more than 100 root hairs.

### 5.4. Gene Expression and GUS Activity Analysis

For analysis of the relative expression levels of the *VLN1* and *VLN4*, total RNA was extracted from root hairs of Col-0 plants using an Easy Pure Plant RNA kit (TransGen Biotech, Beijing, China). Three-day-old Col-0 seedlings grown on the 1/2 MS medium were transferred to 100 μM K^+^ and 200 μM K^+^ medium for 7 d, and then root hairs were collected for RNA extracting. Reverse transcription was carried out by employing the Omniscript Reverse Transcription Kit (TIANGEN Biotech). The transcript levels of gene expression were determined using the Roche Light Cycler 480 system, where *18S* was utilized as an internal reference. All qRT-PCR results were performed with three independent biological replicates. The primer sequences used for qRT-PCR amplification are listed in Table S2.

To analyze the promoter activity of *VLN1* and *VLN4* under low K^+^ treatments, four-day-old *pVLN1*::GUS and *pVLN4*::GUS transgenic seedlings were grown on 100 μM K^+^ and 200 μM K^+^ medium for 10 h before histochemical staining. The acquisition of images was carried out following the previously reported method [15]. Each experiment was conducted three times, resulting in consistent outcomes.

### 5.5. Western Blot Assays

After being cultivated on 1/2 MS medium for 3 days and then transferred to a low K^+^ medium for an additional 7 days, protein extraction was carried out using seedlings harboring the *pVLN1::VLN1-GFP* and *pVLN4::VLN4-GFP* genes. The protein was analyzed using SDS-PAGE, and the method referred to previously reported protocols [36]. The bands of Rubisco were utilized as loading controls.

### 5.6. Quantitative Analysis of Actin Arrays

Fluorescence intensity, skewness, and percentage occupancy are used for analyzing actin filament architecture [37,38]. For the visualization of the actin filaments, *fABD2*-GFP was fused in the Col-0 background. The *vln1-2* and *vln4-1* mutants were hybridized with *fABD2*-GFP. All plants that were homozygous hybrids were chosen for the subsequent experiments. Root hair cells were recorded with lots of overlapping micrographs and analyzed in ImageJ according to the method described previously [39,40]. Concerning these parameters, more than 50 images of root hair cells from no fewer than 30 individual seedlings for each mutant were recorded.

### 5.7. Time-Lapse Imaging of Signal Actin Filament Dynamics

Time-lapse imaging of actin filament dynamics was executed through the methods that had been described previously [24,41]. A specific area of 30 × 30 μm^2^ was chosen within the root cell. Each genotype was assessed by analyzing at least 60 root cells from a minimum of 20 individual seedlings. The analysis was performed using Image J (V1.52g) software.

### 5.8. Potassium Sensitivity Analysis

Three-day-old seedlings of Col-0, *vln1*, and *vln4* mutant and transgenic plants were transferred to 1/2 MS medium and low K^+^ (0, 100, and 200 μM K^+^) medium. The photographs were taken after 13 d of growth, and the survival rate of each genotype was recorded.

### 5.9. Potassium Content Measurement

Three-day-old seedlings of Col-0, *vln1-2*, and *vln4-1* mutant plants were transferred from 1/2 MS medium to the low K^+^ medium with 100 and 200 μM K^+^ for 7 d. The root hair tissues were harvested and dried in the oven at 80 °C for 6 h. Subsequently, they were transferred to Erlenmeyer flasks to undergo the process of heating digestion. Following the completion of heating digestion, the K^+^ content determination involved adjusting the volume to 25 mL and conducting measurements using an Atomic Absorption Spectrophotometer (iCE300/ TAS-986). The results were derived from more than three biological replicates.

## Data Availability

All data are presented in the article and Supplementary Materials.

## Supplementary Materials

The following supporting information can be downloaded at: https://www.mdpi.com/article/10.3390/ijms25168950/s1.
