# Supplementary figures and images for: Dynamics of Actin Filaments Play an Important Role in Root Hair Growth under Low Potassium Stress in Arabidopsis thaliana
Source: Int J Mol Sci. 2024 Aug 16;25(16):8950. doi: 10.3390/ijms25168950 (PMC11354352; doi:10.3390/ijms25168950)

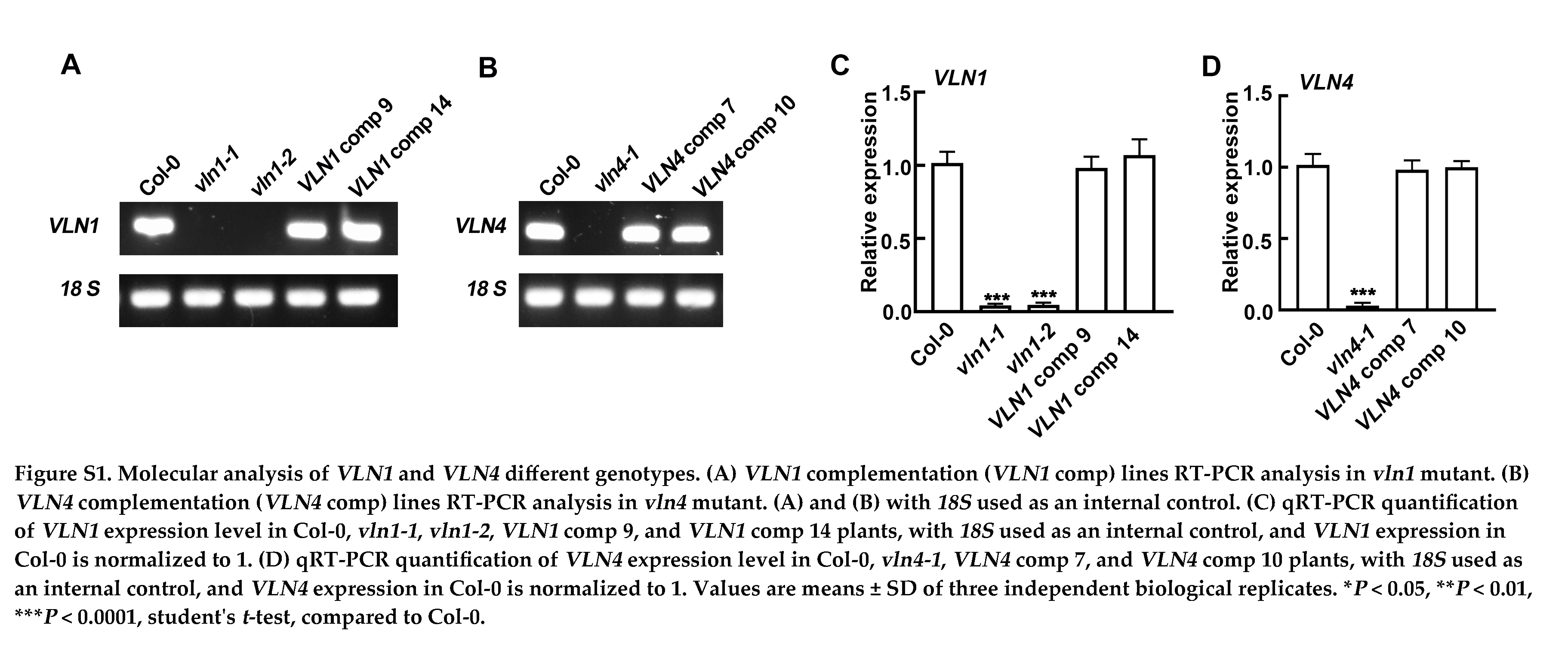

Supplement: Supplementary file 1 [file ijms-25-08950-s001.zip › Figure S1.tif]
